# Texture recognition of pulmonary nodules based on volume local direction ternary pattern

**DOI:** 10.1080/21655979.2020.1807125

**Published:** 2020-08-20

**Authors:** Zhipeng Fan, Huadong Sun, Cong Ren, Xiaowei Han, Zhijie Zhao

**Affiliations:** a School of Computer and Information Engineering, Harbin University of Commerce, Harbin, China; b Key Laboratory of Electronic Commerce and Information Processing of Heilongjiang Province, China

**Keywords:** CAD, random walk, volume local direction ternary pattern, Stacking algorithm

## Abstract

In recent years, the incidence of lung cancer has been increasing. Lung cancer detection is based on computed tomography (CT) imaging of the lung area to determine whether there are pulmonary nodules. And then judge what’s good and what’s bad. However, due to the traditional way of manual reading and lack of experience and other problems. This leads to visual fatigue and misdiagnosis and missed diagnosis. In order to detect pulmonary nodules early and accurately, a new assistant diagnosis method for pulmonary nodules is proposed. Firstly, the image is preprocessed and denoised by median filter, the lung parenchyma is segmented by random walk algorithm and the region of interest is extracted, and then, according to the continuity of the CT slices, the texture feature extraction method of pulmonary nodules based on volume local direction ternary pattern is used to extract the features. Finally, the pulmonary nodules are identified and classified by the assistant diagnosis model of pulmonary nodules based on Stacking algorithm. In order to illustrate the validity of the diagnosis model, the experiments are carried out by cross-validation of ten folds. Experiments using data from LIDC database show that the accuracy, sensitivity and specificity of the proposed method are 82.2%, 85.7%, and 78.8%, respectively. Texture Recognition method based on volume vocal direction ternary pattern is feasible for the identification of pulmonary nodules and provides a reference value for doctor-assisted diagnosis.

## GRAPHICAL ABSTRACT

**Figure:**
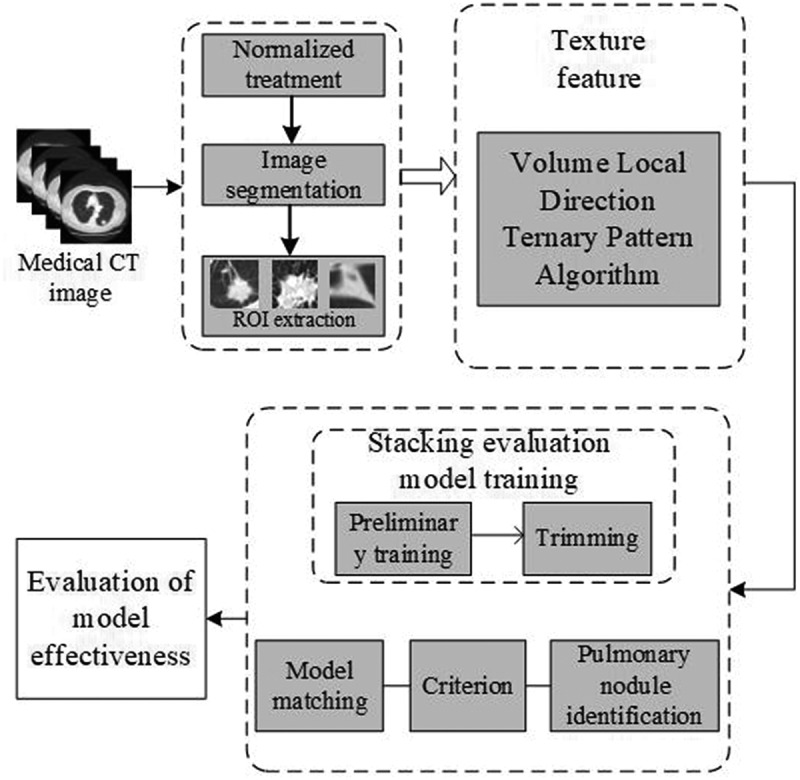

## 1. Introduction

Cancer has become a major problem seriously affecting human health. In the article Global Cancer Statistics 2018 published in CA on 12 September 2018, the incidence and mortality of 36 kinds of cancers in 185 countries were counted. Lung cancer, female breast cancer and colorectal cancer ranked the top three, and cancer mortality in Asia was much higher than that in other regions [1]. In recent years, the number of cancers in China is also increasing. The incidence and mortality of lung cancer rank first [2]. The main reasons include serious air pollution in cities, high smoking population, aging population, difficult to detect in the early stage and difficult to cure in the late stage. Therefore, cancer has alerted people to health problems, and early diagnosis and treatment have become the main factors to reduce mortality.

At present, the main diagnostic methods of lung cancer include X-ray, low-dose spiral CT, nuclear magnetic resonance, biopsy, and so on. According to the National Lung Cancer Screening Experiment of the United States in 2012, low-dose spiral CT can effectively screen lung cancer and reduce mortality [3,4]. Although low-dose spiral CT can improve the survival rate, a large number of CT data and subtle difficult to distinguish nodules cause visual fatigue of doctors, leading to misdiagnosis and missed diagnosis. Aiming at the above problems, computer aided diagnosis technology [5]has been developed continuously in the field of cancer [6,7]. On 20 May 2019, the Google team published an article [8]on lung cancer prediction in Nature-Medicine. This article mainly focused on CT images, established an end-to-end analysis method to predict cancer, and used convolutional neural network to identify nodule. Shihui et al. [9,10] analyzed the research progress of computer-aided diagnosis, mainly from two aspects of machine learning and in-depth learning to explain the assistant diagnosis of lung cancer.

In the process of computer-aided diagnosis, the information of two-dimensional (2D) CT images is usually the main one, and there is no multidimensional information [11,12]. Therefore, multidimensional information makes the diagnosis rate of lung cancer higher. Arai et al. [13] realizes lung image classification through probabilistic neural network of 2D and three-dimensional (3D) local binary pattern. The experimental results show that 3D LBP has higher precision performance than 2D LBP. The classification accuracy of 2D LBP and 3D LBP is 43% and 78% respectively. Wang et al. [14] proposed an algorithm for detecting pulmonary nodules based on the continuity of central points. The region of interest was extracted by super-pixel segmentation method, and the false positive was judged by the deviation degree of a series of central points in ROI. Tong et al. [15] proposed the method of vectorization characteristics of the surface mesh of lung nodules. This method constructs the texture and shape information of pulmonary nodule sphere through 3D reconstruction, and classifies the nodule with deep forest framework based on grid multi-granularity scanning method. The experimental results show that this method is an effective method for 3D feature extraction and vectorization. Although the above methods have been effective, the accuracy of acquisition and classification of 3D information still needs to be improved. Therefore, this paper proposes a new method for pulmonary nodule diagnosis. Firstly, the image is preprocessed, and the lung parenchyma is segmented and the region of interest is extracted by random walk algorithm. Secondly, the texture features of pulmonary nodules are extracted based on volume local direction ternary pattern. Finally, the pulmonary nodules are classified based on Stacking algorithm. The experimental results show that this method can effectively identify and classify pulmonary nodules.

## 2. Related work

With the rapid development of Internet technology, computer has become a hot topic in the medical field. A large amount of medical data makes deep learning technology popular. In 2006, Professor Hinton from the University of Toronto in Canada proposed the deep belief Network in the journal Science [16], making the deep learning technology develop gradually. In 2012, his team made excellent achievements in ImageNet image classification with deep learning technology. Deep learning technology received great attention and research in the field of image research, and set off a wave of science and technology focusing on deep learning. At present, the deep learning techniques applied to the auxiliary diagnosis of lung cancer include convolutional neural network, deep belief network, transfer learning, self-coding, limited Boltzmann machine and its improved algorithm.

Golan et al. [17] proposed a pulmonary tuberous detection system based on deep learning in chest CT images. The system was based on the Lung Image Database (LIDR-IDRI) of the American Cancer Institute and the deep convolutional neural network of the back propagation algorithm to detect and analyze lung nodules from chest CT images of 1018 patients of different sizes and shapes, with a sensitivity of 78.9%. Shi et al. [18] proposed a migration learning method based on deep convolutional neural network (CNN) to detect pulmonary nodules in CT slices. The features of pulmonary nodules were extracted by using the VGG-16 model in the convolutional neural network, and the extracted features were classified and identified by support vector machine. The sensitivity was 87.2%, which was better than other methods for pulmonary nodules identification. Diego et al. [8] established an end-to-end computer-aided lung cancer prediction model, which can predict the overall tumor and pulmonary nodules by using 3D CT lung images, and can solve high false positive and false negative. Shen et al. [19] proposed a multi-crop convolutional neural network for the classification of malignant and suspected lung nodules. This method is to cut out different areas of the convolution feature map, extract and merge features, and realize the classification of suspected nodules. Experimental results show that this method is helpful for the classification of pulmonary nodules malignant tumors and suspected tumors. Patrice et al. [20] proposed a method to distinguish micronodules and non-nodules by using convolutional neural network. This method verifies the classification of micronodules and non-nodules in the convolutional neural network by comparing three different sizes. Experiments show that in the case of 32 × 32 plaque size, the CNN model at two evolutionary layers performs best with an accuracy rate of 88.28%, an AUC of 0.87, an F score of 83.45%, and a sensitivity of 83.82%. Therefore, the proposed CNN model has appropriate image depth and size, which can effectively differentiate pulmonary micronodules (diameter < 3 mm) from non-nodules, and reduce the false positive rate.

The computer-aided diagnosis of pulmonary nodules based on deep learning medical image processing is at the peak of research. Compared with the traditional machine learning medical image processing computer-assisted diagnosis of pulmonary nodules, although the model showed obvious advantages in accuracy, sensitivity, and specificity. However, it has some disadvantages such as poor interpretability of structure, little communication with medicine, high requirement of hardware configuration, complicated training, slow training speed with many parameters and long diagnosis time. Therefore, this paper proposed a CT image recognition method of lung nodules based on the traditional machine learning algorithm. This method can describe the characteristic information of pulmonary nodules in detail and comprehensively, and has high diagnostic efficiency and accuracy, and strong interpretability, which can further improve the level of cancer diagnosis in clinical practice and provide data reference.

## 3. Texture feature extraction of pulmonary nodules based on volume local direction ternary pattern

Pulmonary nodule texture feature extraction is one of the feature extraction methods to identify pulmonary nodules. Its main purpose is to show some texture changes and trends of pulmonary nodules in numerical form. This paper presents a new method for extracting texture features of pulmonary nodules: a texture feature extraction algorithm based on volume local direction ternary pattern. Firstly, the lung CT image is preprocessed, including CT image normalization and denoising, lung parenchyma segmentation and region of interest extraction; Secondly, the volume local ternary pattern extraction is carried out for ROI; Finally, the directional ternary statistics is used to compute the volume local ternary pattern, forming the eigenvector. The eigenvector is normalized.

### 3.1. CT image preprocessing

#### 3.1.1. CT image normalization and denoising

Due to the great differences in acquisition and reconstruction protocols of different imaging devices, lack of uniform standards, and the format of acquired DICOM lung CT images and noise introduced by external factors during scanning and so on, it is not suitable for CT images to be processed directly. Therefore, before image segmentation, CT image should be normalized and denoised. In this paper, we use Sant Dicom Viewer software to process CT image into gray-scale image with uniform window width, window length and image size of 512*512. Then we use median filter to denoise image with MATLAB software, so that CT image can effectively retain texture details, improve image clarity, and lay a foundation for texture feature extraction.

**Figure 1.**
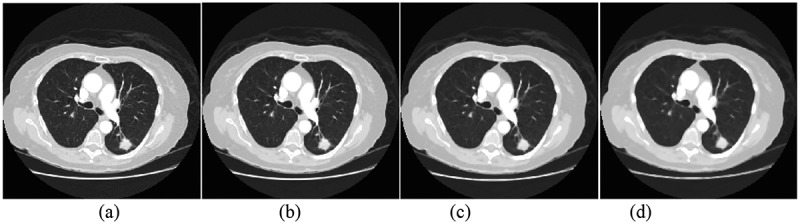
Median filtering: (a) original image (b)3 × 3 template (c)5 × 5 template (d)7 × 7 template.

In the process of computerized tomography scanning, the autonomous or involuntary movement of human body will lead to artifacts of the detected object and blur the detected object. Therefore, it is necessary to denoise the lung CT image before processing. This paper mainly uses median filtering method to denoise image. The experimental results are shown in Figure 1.

In the median filtering of Figure 1, the 3 × 3, 5 × 5 and 7 × 7 templates are used to filter. The experimental results show that with the increase of the template, the image details become more and more blurred, and the image is clearer by using 3 × 3 template, so this paper uses 3 × 3 template to denoise. Median filtering can not only effectively remove the noise in the image, but also retain the edge information of the image.

#### 3.1.2. Segmentation of lung parenchyma and extraction of region of interest

**Figure 2.**
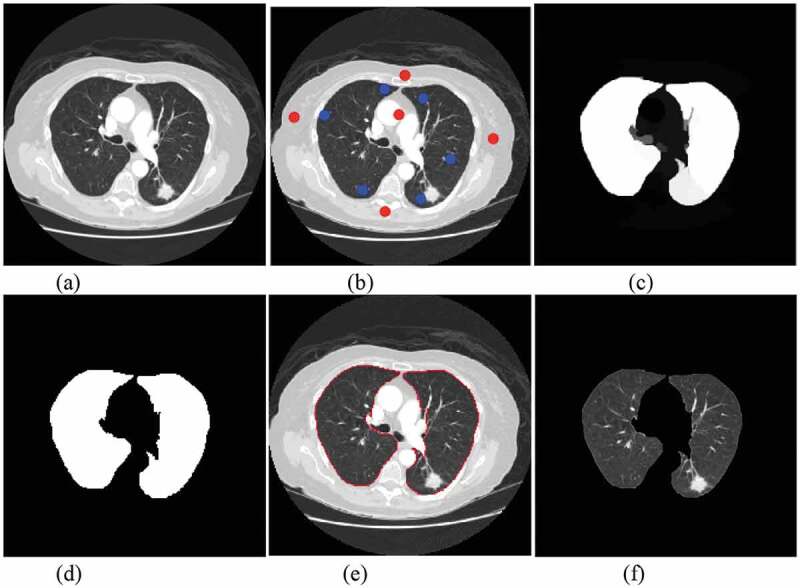
Pulmonary parenchymal segmentation: (a)original image. (b) front and background seed settings (c) front probability calculation (d) binarization (e) segmentation boundary (f) pulmonary parenchymal segmentation.

In the process of pulmonary nodule detection, the segmentation of lung parenchyma will affect the accuracy, so the correct segmentation algorithm can improve the identify accuracy. Singadkar et al. [21] proposed an unsupervised and fully automatic lung parenchyma segmentation method based on the spatial interaction between adjacent pixels and the constrained nonnegative matrix. Zhang et al. [22] proposed a spatial clustering method based on super-pixel and density (DBSCAN) to segment lung noduleimage sequence. Wang et al. [23] proposed a method based on Random Walk for automatic segmentation of lung parenchyma. Considering the characteristics of lung parenchyma, this paper uses random walk algorithm to segment the lung parenchyma. The algorithm mainly includes: setting seed points of front and background; calculating foreground probability; image binarization; image segmentation boundary; image mask processing. Figure 2 is the result of segmentation of lung parenchyma by random walk algorithm.

**Figure 3.**
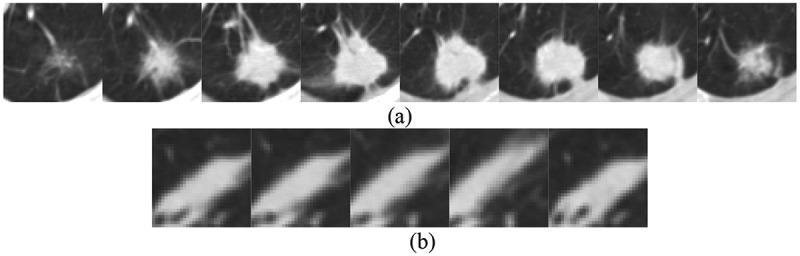
ROI extraction results: (a) pulmonary nodules (b) suspected nodules.

ROI extraction is an important process in medical image processing. It extracts regions of interest from lung parenchyma. The results of ROI extraction can be divided into two categories: pulmonary nodules and suspected nodules. Figure 3(a) shows CT images of pulmonary nodules and Figure 3(b) shows CT images of suspected nodules.

### 3.2. Feature extraction framework based on volume local direction ternary patterns

At present, the existing methods of pulmonary nodule texture feature extraction mainly focus on 2D images. Texture feature ignores the spatial distribution of texture and presents some texture information, which leads to the low identify rate of pulmonary nodules. In this paper, a texture feature extraction algorithm for pulmonary nodules based on volume local direction ternary pattern is proposed. The steps of the algorithm are as follows:

Input: Pulmonary nodule image sequence; Output: Eigenvector

Step 1: Pulmonary nodule image sequence is sorted in order to form a 3D pulmonary nodule.

Step 2: Local pattern extraction of adjacent sections of pulmonary nodules based on VLBP model [24].

Step 3: The local adaptive threshold based on normal function is used to calculate the local pattern extracted from step 2.

Step 4: The local ternary pattern calculated by step 3 is used to calculate the three-valued probability in all directions with the central pixel as the center.

Step 5: Normalize the three-value probability of each direction as the characteristic vector of the pulmonary nodule.

**Figure 4.**
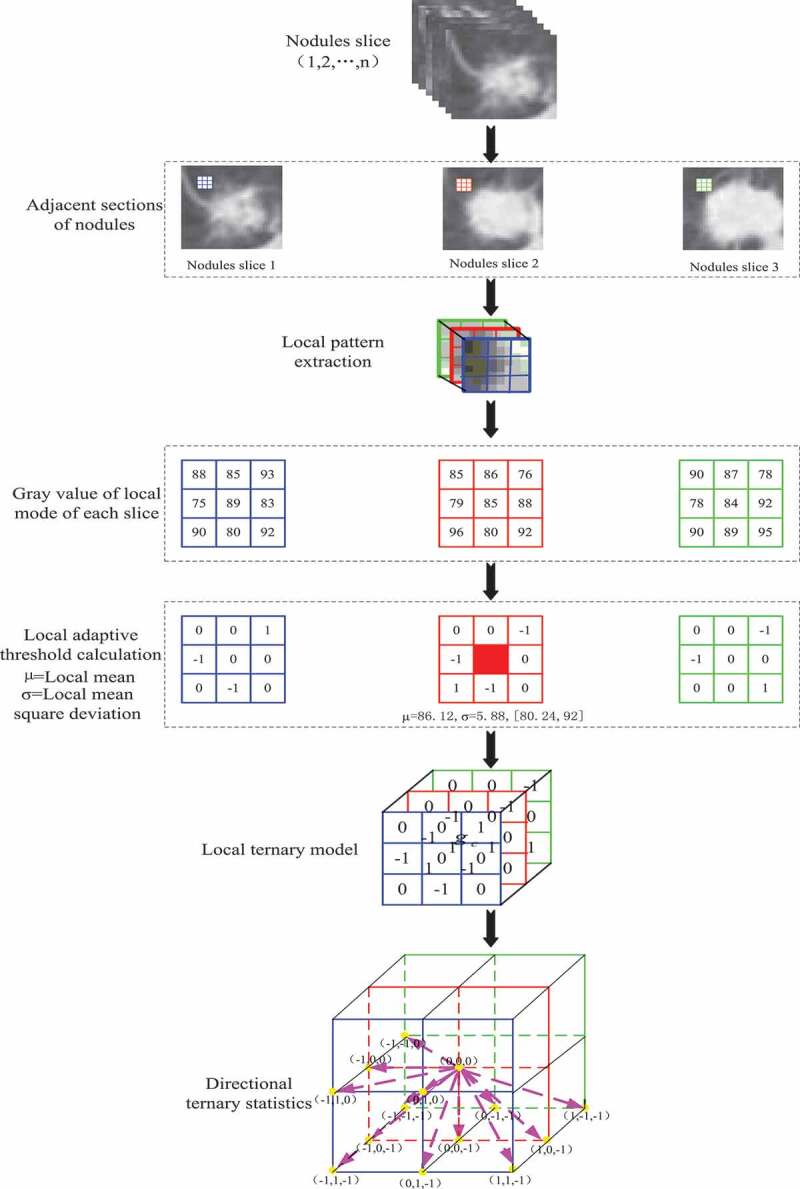
Texture feature extraction of pulmonary nodules.

Figure 4 depicts the whole extraction process of pulmonary nodule texture features under volume local direction ternary pattern. Firstly, the preprocessed pulmonary nodule slices are discharged one by one in order; secondly, the adjacent slices are selected to form a 3*3*3 pixel matrix with the central pixel of a single pixel and its adjacent 26 pixels, i.e. the volume local patterns; the pixel values in the local patterns obey the normal distribution, and their thresholds are determined by the expectations and variances of the normal distribution characteristics to form the volume local ternary patterns. Then, the number of occurrences of – 1, 0, +1 of central pixels in 13 directions is counted according to the obtained volume local ternary patterns, forming the 13 *3 dimension feature vectors. The feature vectors of all local patterns are added and normalized to form the final feature vectors.

### 3.3. Volume local direction ternary pattern extraction algorithms

#### 3.3.1. Construction of local patterns

Based on VLBP, a 3D reconstruction of adjacent sections of pulmonary nodules is carried out in this paper. The construction process is as follows:

1. The selected pulmonary nodules were arranged in order of slices 1, 2, …, n, which showed the information of pulmonary nodules in time dimension.
2. According to the sequence of slices, three adjacent lung nodules are selected. Based on the central slice, a neighborhood pixel matrix of 3 × 3 × 3 is formed by selecting each pixel as the center in the slice plane of pulmonary nodules, i.e. the pixel matrix formed by the central pixel and 26 neighborhood pixels around it. The 3 × 3 × 3 pixel matrix is a local pattern.
3. According to the formation mode of the pixel matrix in (2), all lung nodule slices were constructed with each pixel as the center, and the neighborhood pixel matrix was constructed, that is, the neighborhood pixel matrix of 3 × 3 × 3 was formed. Assuming that a pulmonary nodule has n slices and the pixel size of each slice is m × m, the number of final local patterns of the pulmonary nodule is $\left(n-2\right)\left(m-2\right)\left(m-2\right)$.

#### 3.3.2. Local adaptive threshold calculation

Zhao et al. [24] used dynamic texture feature to recognize facial expression based on VLBP. V was defined as dynamic texture feature and as shown in formula (1) (2). Where P was the number of neighborhood pixels of the central pixel, L was the time interval between the previous frame and the posterior frame, and $g_{t_{c}},c$ was the central pixel value of the local pattern.

(1) $$\begin{array}{rl} & V=v\left(g_{t_{c}}-L,c,g_{t_{c}}-L,0,\ldots ,g_{t_{c}}-L,P-1,g_{t_{c}},\right.\\ & \;\;\;\;\;\;\;\;\;\;\;\;c,g_{t_{c}},0,\ldots ,g_{t_{c}},P-1,g_{t_{c}}+L,0,\ldots ,g_{t_{c}}\\ & \left.\;\;\;\;\;\;\;\;\;\;\;\;+L,P-1,g_{t_{c}}+L,c\right) \end{array}$$

(2) $$\begin{array}{rl} & VLBP=v\left(s\left(g_{t_{c}}-L,c-g_{t_{c}},c\right),s\left(g_{t_{c}}-L,0-g_{t_{c}},c\right)\right.,\ldots ,\\ & \;\;\;\;\;\;\;\;\;\;\;\;\;\;\;\;\;\;\;\;\;\;s\left(g_{t_{c}}-L,P-1-g_{t_{c}},c\right),s\left(g_{t_{c}},0-g_{t_{c}},c\right),\ldots ,\\ & \;\;\;\;\;\;\;\;\;\;\;\;\;\;\;\;\;\;\;\;\;\;s\left(g_{t_{c}},P-1-g_{t_{c}},c\right),s\left(g_{t_{c}}+L,0-g_{t_{c}},c\right),\ldots ,\\ & \left.\;\;\;\;\;\;\;\;\;\;\;\;\;\;\;\;\;\;\;\;\;\;s\left(g_{t_{c}}+L,P-1-g_{t_{c}},c\right),s\left(g_{t_{c}}+L,c-g_{t_{c}},c\right)\right) \end{array}$$

Where

(3) $$s\left(x\right)=\left\{\begin{array}{c} 1,x\geq 0\\ 0,x<0 \end{array}\right.$$

Tan et al. [25] proposed LTP on the basis of LBP. It calculates texture features by setting appropriate thresholds. Therefore, this paper combines this method to calculate the volume local direction ternary mode. Volume local direction ternary pattern is a method for calculating 3D local texture features based on the texture orientation and change of pulmonary nodules in time dimension. The main idea of this pattern is to illustrate the local change characteristics of the central pixel on the pulmonary nodule according to any adjacent pulmonary nodule slice, the change relationship between the arbitrary pixel centered on the central slice and the 26 adjacent pixels based on normal function. It can not only reflect the change information of pixels in spatial dimension, but also add the change information of pulmonary nodule texture in temporal dimension. In this method, the pixel values of all local patterns obey normal distribution, so the mean values of all local patterns are the central pixels, and the variance of all domain pixels in the local patterns are the adaptive thresholds. Through the central pixel, adaptive threshold and neighborhood pixel, formula (4) calculates and compares the results, and assigns the results to the neighborhood pixels with which it participates in the calculation.

(4) $$\vec{f}\left(g_{p},\mu ,\sigma ,k\right)=\left\{\begin{array}{c} +1,\;g_{p}\;>\;\mu +k\sigma \\ 0,\;|g_{p}-\mu |\leq k\sigma \\ -1,\;g_{p}\;<\;\;\mu -k\sigma \end{array}\right.$$

Where In the formula (4), μ denotes the central pixel which is worth calculating according to the 26 neighborhood pixel values and the central pixel values; $g_{p}$ denotes the neighborhood pixel; σ denotes the adaptive threshold which is obtained by calculating the mean square error based on the 26 neighborhood pixel values; k denotes the threshold coefficients, which are 1, 0.43 and 0.675, respectively; $\vec{f}\left(x\right)$ denotes the relationship between the central pixel and the neighborhood pixel.

**Figure 5.**
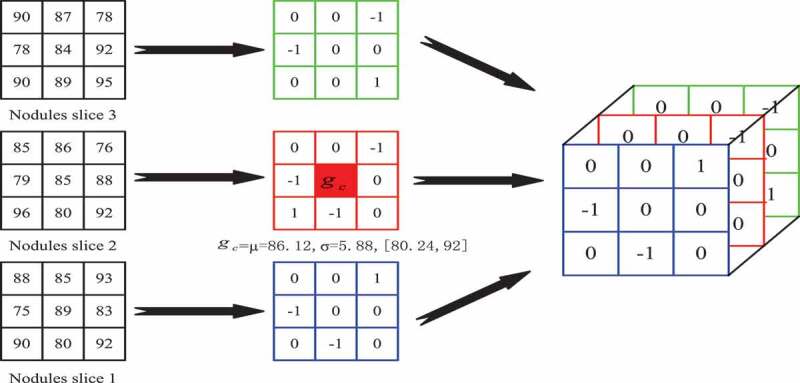
Extraction process of volume local ternary patterns.

Where In the formula (4), when the neighborhood pixel $g_{p}$ is greater than the sum $\left(\mu +k\sigma \right)$ of the center pixel and the k-fold offset pixel, the value of $g_{p}$ is assigned to + 1 in the volume local ternary patterns; when the neighborhood pixel $g_{p}$ is smaller than the difference $\left(\mu -k\sigma \right)$ of the center pixel and the k-fold offset pixel, the value of $g_{p}$is assigned to -1 in the volume local ternary patterns; When the neighborhood pixel $g_{p}$ is between the sum and difference ($\left[\mu -k\sigma ,\mu +k\sigma \right]$) of the central pixel and k-fold offset pixel, the value of $g_{p}$ is assigned to 0 in the volume local ternary patterns. The extraction process of volume local ternary patterns is shown in Figure 5.

Figure 5 is the process of extracting and calculating the volume local ternary patterns. Firstly, the mean μ of the local pattern is calculated according to all the pixels in the local pattern, i.e. the central pixel value μ=86.12; Secondly, the 26 neighborhood pixel values $g_{c}$ and the central pixel value μ are computed to get the fixed threshold σ, i.e. the bias pixel values σ=2. In clockwise direction, when the threshold coefficient K is 1, when the neighborhood pixel is 93, it is not between its threshold range [80.24,92] and is greater than 92, the position $g_{p5}$ is coded as +1. When the neighborhood pixel is 85, it is between its threshold range [80.24,92], the position $g_{p4}$ is coded as 0; when the neighborhood pixel is 75, it is not between its threshold range [80.24,92] and is less than 80.24, the position $g_{p2}$ is coded as – 1; and by analogy, all coding triple values of the local patterns are calculated.

Formula for calculating local adaptive threshold σ:

(6) $$\sigma =\sqrt{\frac{1}{N-1}\sum_{i=1}^{N-1}{\left(x_{i}-\mu \right)}^{2}}$$

Where In the formula (6), $x_{i}$ denotes the ith pixel value, μ denotes the mean of local pattern pixels, and *N* denotes the number of local mode pixels with a value of 27.

#### 3.3.3. Volume local direction ternary pattern

**Figure 6.**
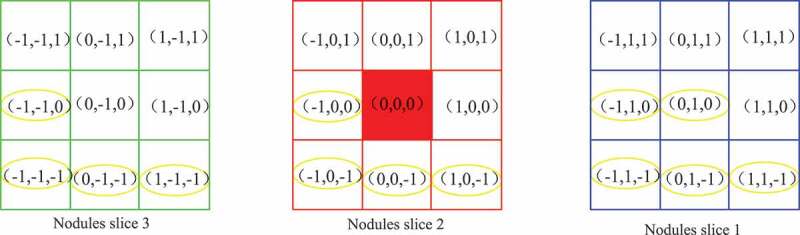
Two-dimensional planar pattern of central pixel.

Volume local ternary pattern is a ternary pattern composed of -1,0,1. In traditional volume local binary pattern, these binary values are coded according to certain rules and given corresponding weights, and finally the eigenvectors are formed in the form of histograms. In this paper, we propose a feature calculation pattern for calculating the volume local direction ternary, which combines the trends of change of the pulmonary nodule texture in spatial and temporal dimensions and the concept of direction in the 3D gray level co-occurrence matrix [26]. The method applies the concept of direction, i.e. taking the texture of pulmonary nodules as the gray level change in a certain direction, using the volume local ternary patterns and taking the central pixel as the center to carry out the three-value probability statistics in all directions. The thirteen directions of θ are (0 1 0; −1 1 0; −1 0 0; −1 −1 0; 0 1–1; 0 0 −1; 0 −1 −1; −1 0 −1; 1 0 −1; −1 1 −1; 1 −1 −1; −1 −1 −1; 1 1 −1). The 2D plan of each direction of the central pixel is shown in Figure 6.

**Figure 7.**
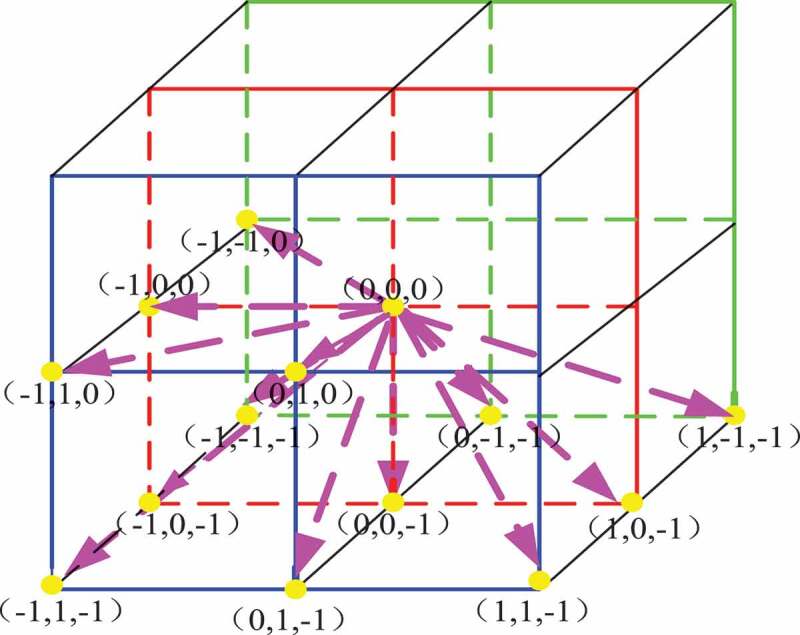
Stereo direction pattern of central pixel.

In the 2D plane pattern of the central pixel in Figure 6, the red region is the central pixel of the volume local ternary pattern, and the yellow circle represents the thirteen directions of the central pixel. Volume local direction ternary pattern is based on the volume local ternary pattern to calculate the ternary values – 1, 0, +1 appearing in the thirteen directions, forming a 13 × 3 dimensional feature vector. The stereo plane pattern of the central pixel is shown in Figure 7.

The eigenvector of the local direction ternary pattern proposed in this paper is the statistical calculation of all the local ternary patterns of a single pulmonary nodule, and the sum of the ternary of each direction is connected to form the eigenvector. The calculation formula is shown in (7).

(7) $$H(VLDTP)=\sum_{N_{1}-1}^{i=2}\sum_{N_{2}-1}^{j=2}\sum_{N_{3}-1}^{k=2}f\left(LTP\left(i,j,k\right),\theta \right)$$

Where In the formula, $N_{1}\times N_{2}\times N_{3}$ denotes the size of 3D image, LTP local ternary mode, $\left(i,j,k\right)$ denotes the pixels in row i column j layer k of the image, θ denotes all directions of $\left(i,j,k\right)$. The above eigenvectors are normalized:

(8) $$H\;(NVLDTP)=\frac{H\;(VLDTP)}{\left(N_{1}-2\right)\left(N_{2}-2\right)\left(N_{3}-2\right)}$$

## 4. Pulmonary nodule recognition based on Staking algorithm

Stacking algorithm [27–29] is a kind of ensemble learning method, which combines multiple learners in a nonlinear way to achieve better learning effect. Firstly, the feature data sets are divided randomly, and the feature data sets are independent of each other. and $x_{n}$ is the sample and $x_{n}\in \left[0,1\right]$, and $y_{n}$ is the label and $y_{n}=\left\{1,0\right\}$. The formula is defined as follows:

(9) $$\begin{array}{rl} & D=\left\{\left(x_{1},y_{1}\right),\left(x_{2},y_{2}\right),\ldots ,\left(x_{n},y_{n}\right)\right\},\\ & \;\;\;\;\;\;D_{1}\cap ,D_{2}\cap ,\ldots ,\cap D_{n}=\varphi \end{array}$$

Then, the data set is input into each learner $h_{1},h_{2},\ldots ,h_{m}$ to form a primary learner h. $\Phi =\left\{\Phi_{1},\Phi_{2},\ldots ,\Phi_{m}\right\}$ is the output of primary classifier and $\Phi \in \left\{1,0\right\}$. The formula is defined as follows:

(10) $$h=\left\{\begin{array}{c} h_{1}:D_{1}\to \Phi_{1}\\ h_{2}:D_{2}\to \Phi_{2}\\ \;\;\;\;\;\;\;\;\;\;\;\;\;\vdots \\ h_{m}:D_{m}\to \Phi_{m} \end{array}\right\},\;\;\;\Phi_{1}\cap^{\Phi_{2}},\ldots ,\cap^{\Phi_{m}}=\varphi$$

(11) $$\phi_{j}=\left\{h\left(d_{i}\right)|d_{i}\in D_{m}\right\},\;\;\;\;\phi_{j}\in \Phi$$

Finally, the results of K-fold training of the primary learner h are input into the secondary learner $h^{\prime }$ as a new data set $D^{\prime }=\Phi =\left\{\Phi_{1},\Phi_{2},\ldots ,\Phi_{m}\right\}$. The formula is defined as follows:

(12) $$h^{\prime }=\left\{h^{\prime }:D^{\prime }\to \Phi^{\prime }\right\}$$

(13) $$\phi_{j}^{\prime }=\left\{h^{\prime }\left({d^{\prime }}_{i}\right)|{d^{\prime }}_{i}\in D^{\prime }\right\},\phi_{j}^{\prime }\in \Phi^{\prime }$$

**Figure 8.**
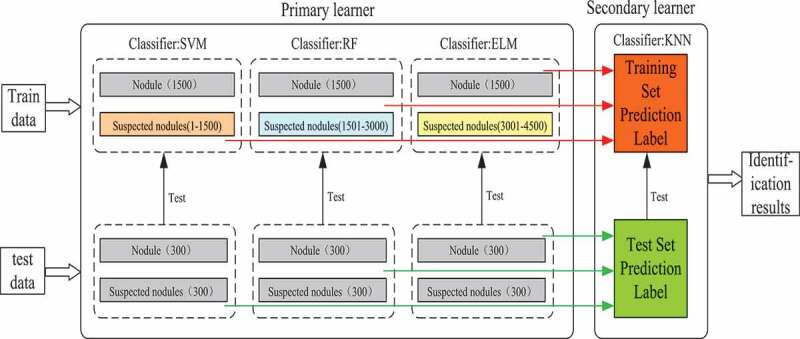
Assistant diagnosis model of pulmonary nodules based on Staking algorithm.

Based on the characteristics of texture feature extraction, an assistant diagnosis model of pulmonary nodules based on Stacking algorithm is constructed in this paper. Firstly, the unbalanced samples are divided into independent disjoint sets; then, the primary learners are trained and tested by classifiers SVM, RF and ELM; secondly, the output of the primary learners is used as the input of the secondary learners, and the KNN is used for training and testing; finally, the model is evaluated according to the evaluation index and the receiver operator characteristics curve. Figure 8 is an assistant diagnosis model of pulmonary nodules based on Staking algorithm.

Although there are many ensemble methods, Stacking algorithm is better than any single model for heterogeneous ensemble. Therefore, an assistant diagnosis model of pulmonary nodules based on Stacking algorithm is proposed. The diagnostic process of this model is as follows:

1. Aiming at the imbalance of training samples, the project achieves sample balance by changing the distribution of training samples (i.e. training set partition), and divides training samples and test samples into disjoint subsets based on Stacking algorithm.
2. The primary learner is composed of support vector machine, nearest neighbor classification and extreme learning machine to predict the label of training set and test set.
3. The training set and test set labels obtained by the primary learner are used as the input of the secondary learner, and the final classification results are obtained by learning with random forest.
4. The final classification results are analyzed by the evaluation index and the receiver operator characteristics curve, and the final evaluation of the model is obtained.

## 5. Experiments and analysis

### 5.1. Introduction of experimental data

The experiment was carried out using the lung image database [30] (LIDC-IDRI) collected by the National Cancer Institute of the United States. There were 1010 subjects, 1018 research cases and 244,527 images in this database. The 244 527 image files in the database are all standard format Dicom of medical images. Each CT image has 512 × 512 pixels. In addition to this pixel information, there are some auxiliary metadata information, including patient ID, image location, slice distance, resolution, etc.A total of 6937 experimental samples were screened out, including 1884 pulmonary nodules and 5053 suspected nodules.

### 5.2. Data characteristic analysis

For medical images, the proportion of lesion images is very small, and the proportion of lesion images and normal medical images is about 1:1000 or more. The lung image is no exception. The ratio of lesion image to normal image is about 1:3. When the prior probability is small, all data will tend to be judged as normal image, and the correct rate will be more than 90%. In this paper, we extract texture features of pulmonary nodules and suspected nodules based on volume local direction ternary patterns. Because of the imbalance of experimental samples, the amount of texture feature data extracted also has imbalance between positive and negative samples, i.e. 1884 samples of pulmonary nodules and 5053 samples of suspected nodules. According to the dividing steps of training set, firstly the reasonable distribution ratio of positive and negative samples is calculated: pulmonary nodules: suspected nodules = 1:3; secondly, through the number of rare samples (pulmonary nodules), a large number of samples (suspected nodules) were randomly divided into three independent sample subsets; finally, three balanced subsets of samples were formed by combining the lung nodule samples with the suspected nodule samples: 3 x (1500 pulmonary nodules, 1500 suspected nodules), waiting for the training of classifier.

### 5.3. Evaluation criteria

In the computer aided diagnosis system, the assisted diagnosis model is mainly evaluated by the evaluation indexes [31] such as Accuracy, Sensitivity, Specificity, Matthews correlation coefficient (MCC) and F1 score.

(14) $$Accuracy=\frac{TP+TN}{TP+TN+FP+FN}\times 100\%$$

(15) $$Sensitivity=TPR=\frac{TP}{TP+FN}\times 100\%$$

(16) $$Specificity=TNR=\frac{TN}{TN+FP}\times 100\%$$

(17) $$MCC=\frac{TP\times TN-FP\times FN}{\sqrt{\left(TP+FP\right)\left(TP+FN\right)\left(TN+FP\right)\left(TN+FN\right)}}$$

(18) $$F1=2\frac{Sensitivity\times \mathrm{Pr}ecision}{Sensitivity+\mathrm{Pr}ecision}$$

In order to more intuitively represent the performance and diagnostic effect of the classifier, the receiver operator characteristics curve is also used as the evaluation criterion. Receiver operator characteristics curve takes area under curve as a measure of classifier performance. Its abscissa is false positive rate (1-specificity), and its ordinate is true positive rate (sensitivity).It combines sensitivity and specificity to accurately reflect the relationship between the two classifiers. The closer the area under the curve is to 1, the better the performance of the classifier will be. When the area under the curve is 0.5, the failure of the classifier will be indicated. The closer the curve is to the upper left corner, the higher the accuracy of the classifier will be.

### 5.4. Results and analysis

#### 5.4.1. Selection experiments of different texture features and threshold parameters

In order to verify the validity of texture feature extraction method, 3000 samples were selected for experiment, including 1500 pulmonary nodules and 1500 suspected nodules, and 10 fold cross validation was used to fully demonstrate the validity of the model. Samples were divided into 10 samples, one of which was used as training set for each model training, and 10 training sessions were conducted to find the average value as the performance of classification model. Evaluation value. In this experiment, four classifiers are used: Support Vector Machine, Nearest Neighbor Classification, Random Forest and Extreme Learning Machine to verify the extracted texture features. In the parameter selection of classifier, the support vector machine chooses the Gauss Radial Basis Function (RBF), the nearest neighbor classifier uses the Euclidean parameter with the neighborhood number 2, the distance is the Euclidean parameter, the random forest uses 100 decision trees, the hidden layer of the extreme learning machine is 900, and the activation function is Sigm. The above parameters have been verified by experiments to obtain the best results.

In this paper, four classifier models, Support Vector Machine, Nearest Neighbor Classification, Random Forest and Extreme Learning Machine, are used to analyze the selection of threshold coefficient K based on local adaptive threshold of volume local direction ternary pattern and 3D gray level co-occurrence matrix. The threshold coefficient K is 1, 0.43 and 0.675. In order to more intuitively illustrate the performance of extracted texture features on classifier, the accuracy, sensitivity, specificity, Matthews correlation coefficient, F1 score and other evaluation indicators are used to evaluate. Tables 1–4 are the classification results of texture features of volume local direction ternary pattern and 3D gray level co-occurrence matrix with different threshold coefficients under four classifiers, respectively.

**Table 1.** Classifier SVM classification results.

Evaluation Criteria Texture features	Accuracy	Sensitivity	Specificity	MCC	F1
VLDTP, K = 1	0.807	0.848	0.765	0.616	0.814
VLDTP, K = 0.43	0.801	0.845	0.758	0.606	0.809
VLDTP, K = 0.675	0.800	0.852	0.748	0.604	0.810
3D-GLCM	0.774	0.780	0.769	0.549	0.775

**Table 2.** Classifier KNN classification results.

Evaluation Criteria Texture features	Accuracy	Sensitivity	Specificity	MCC	F1
VLDTP, K = 1	0.752	0.804	0.700	0.524	0.764
VLDTP, K = 0.43	0.747	0.790	0.707	0.512	0.757
VLDTP, K = 0.675	0.735	0.780	0.689	0.486	0.744
3D-GLCM	0.746	0.755	0.740	0.511	0.746

**Table 3.** Classifier RF classification results.

Evaluation Criteria Texture features	Accuracy	Sensitivity	Specificity	MCC	F1
VLDTP, K = 1	0.819	0.858	0.778	0.640	0.826
VLDTP, K = 0.43	0.811	0.844	0.779	0.624	0.817
VLDTP, K = 0.675	0.801	0.844	0.757	0.604	0.809
3D-GLCM	0.800	0.807	0.793	0.600	0.801

**Table 4.** Classifier ELM classification results.

Evaluation Criteria Texture features	Accuracy	Sensitivity	Specificity	MCC	F1
VLDTP, K = 1	0.761	0.836	0.686	0.528	0.777
VLDTP, K = 0.43	0.743	0.803	0.683	0.490	0.757
VLDTP, K = 0.675	0.739	0.821	0.656	0.484	0.758
3D-GLCM	0.664	0.680	0.647	0.328	0.668

**Figure 9.**
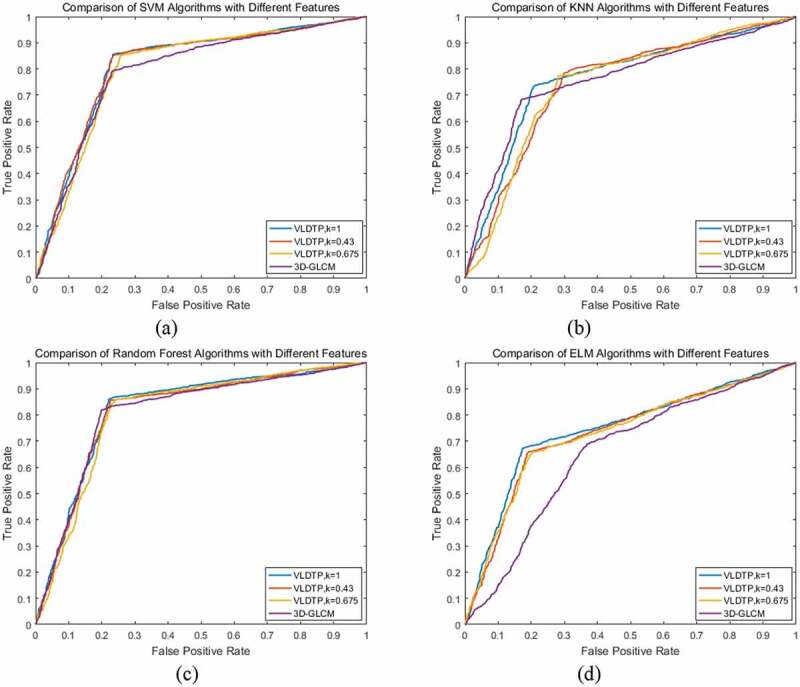
ROC curve of pulmonary nodules classification results.

From Tables 1–4, we can see that when the threshold coefficient K of the local adaptive threshold of volume local direction ternary pattern is 1, all the other evaluation indexes except specificity are the highest on classifier support vector machine, nearest neighbor classification and random forest. The receiver operator characteristics curve is shown in Figure 9(a–c), and the area under each curve is very close, but the threshold is the highest. However, in terms of specificity, the curve of the 3D gray level co-occurrence matrix is close to the upper left, so the misdiagnosis rate is low. On the classifier extreme learning machine, when the threshold coefficient K of the local adaptive threshold of the volume local direction ternary pattern is 1, each evaluation index achieves the maximum value. The receiver operator characteristics curve is shown in Figure 9(d), which shows that the results are consistent with Table 4, and the rate of missed diagnosis and misdiagnosis is low. Based on the above analysis, when the threshold coefficient K of the local adaptive threshold of volume local direction ternary pattern is 1, the texture features of volume local direction ternary pattern get different classification results under four classifiers, but they all get good classification results under each classifier. Therefore, when the threshold coefficient *K* is 1, the texture features extracted from the volume local direction ternary pattern have better identification effect than the texture features of the 3D gray level co-occurrence matrix, which proves that the proposed algorithm is effective in the identification and classification of pulmonary nodules and improves the diagnosis rate.

#### 5.4.2. Experimental results of pulmonary nodule recognition based on Stacking algorithm

In order to verify the validity of the Stacking algorithm-based lung nodule diagnosis model for lung nodule identification, 6937 samples were tested. According to the method of training set partition and random sampling without placing back, training set samples were obtained: 3 x (1500 pulmonary nodules, 1500 suspected nodules), test set samples: 1 x (300 pulmonary nodules, 300 suspected nodules), which were validated by 10-fold cross validation. In this paper, pulmonary nodules are classified by the Staking-based diagnostic model for pulmonary nodules. The model consists of two levels of learners: Leve1-SVM, Leve1-RF, Leve1-ELM, and Leve2-KNN. Tables 5 and 6 are the training and testing results of each classifier at different network levels.

**Table 5.** Training results of classifiers at different levels.

Evaluation Criteria Classifier	Accuracy	Sensitivity	Specificity	MCC	F1
Leve1-SVM	0.848	0.802	0.756	0.608	0.811
Leve1-KNN	0.738	0.763	0.787	0.527	0.757
Leve1-RF	0.856	0.815	0.775	0.633	0.823
Leve1-ELM	0.830	0.748	0.673	0.502	0.765
Leve2-KNN	0.931	0.918	0.905	0.836	0.919

**Table 6.** Test results of classifiers at different levels.

Evaluation Criteria Classifier	Accuracy	Sensitivity	Specificity	MCC	F1
Leve1-SVM	0.847	0.795	0.744	0.594	0.806
Leve1-KNN	0.702	0.731	0.760	0.463	0.723
Leve1-RF	0.855	0.802	0.748	0.608	0.812
Leve1-ELM	0.828	0.743	0.656	0.494	0.764
Leve2-KNN	0.879	0.831	0.783	0.665	0.839

It can be seen from Table 5 that in the training set, under each classifier of the base classifier, the accuracy of the random forest is the highest: 0.856, while under the meta classifier, the accuracy reaches 0.931, increasing by nearly 8%; under each classifier of the base classifier, the sensitivity of the random forest is the highest: 0.815, while under the meta classifier, the sensitivity reaches 0.918, increasing by nearly 10%; Under each classifier of the base classifier, the specificity of k-nearest neighbor classification is the highest: 0.787, while under the meta classifier, the specificity reaches 0.905, increasing nearly 12 percentage points; under each classifier of the base classifier, the Matthews correlation coefficient of the random forest is the highest: 0.633, while under the meta classifier, the Matthews correlation coefficient reaches 0.836, increasing nearly 20 percentage points; under the base classifier, the Matthews correlation coefficient reaches 0.836, increasing nearly 20 percentage points Under each classifier, the highest F1 score of random forest is 0.823, while under the meta classifier, the F1 score reaches 0.919, increasing nearly 9 percentage points. According to the above analysis, in the lung nodule image recognition based on stacking algorithm, all kinds of evaluation indexes of training set are improved, not only the accuracy of judgment is improved, but also the misdiagnosis rate and missed diagnosis rate are reduced.

Table 6 shows the test results under the stacking evaluation model. It can be seen from the table that under each classifier of the base classifier, the accuracy of random forest is the highest: 0.855, while under the meta classifier, the accuracy reaches 0.879, increasing by nearly 2.4%; under each classifier of the base classifier, the sensitivity of random forest is the highest: 0.802, while under the meta classifier, the sensitivity is the highest Under the basic classifier, the specificity of k-nearest neighbor classification is the highest: 0.760, while under the meta classifier, the specificity is 0.783, which is increased by nearly 2.3%; under the basic classifier, the Matthews correlation coefficient of random forest is the highest: 0.608, while under the meta classifier, the Matthews correlation coefficient is 0 665, an increase of nearly 5.7%; under each classifier of the base classifier, the F1 score of random forest is the highest: 0.812, while under the meta classifier, the F1 score reaches 0.839, an increase of nearly 2.7%. According to the above analysis, in the lung nodule image recognition based on stacking algorithm, all kinds of evaluation indexes of the test set have been improved, which proves that this method can effectively classify the lung nodule image.

**Figure 10.**
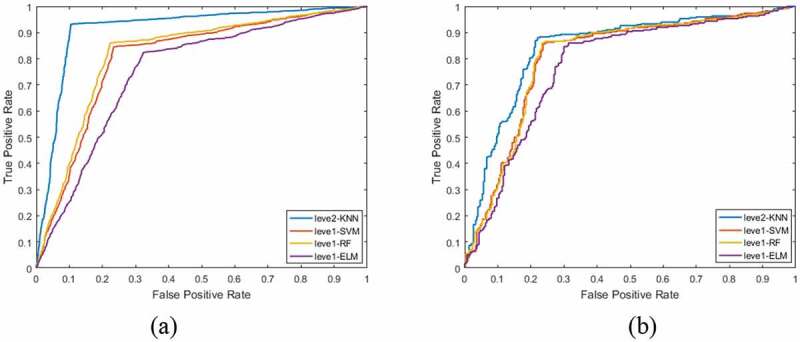
Performance comparison of two level learners.

Figure 10 is a performance comparison of two-level learners. Figure 10(a) is a comparison of ROC curves of each classifier under the primary learner and under the secondary learner in the training set. It can be seen that the area under the ROC curve of the primary learner is not only the largest, but also the curve is close to the upper left. Figure 10(b) is a comparison of ROC curves of primary learners and secondary learners in test set.

#### 5.4.3. Verification experiment with other papers

In order to verify the effectiveness of this method for the identification of pulmonary nodules, this paper compares the accuracy, sensitivity, specificity and F1 score with other methods. Table 7 shows the comparison of different methods for pulmonary nodule recognition. From the data analysis in the table, we can see that the accuracy, sensitivity, specificity and F1 score of this method are the highest, respectively 0.931, 0.918, 0.905 and 0.919. Therefore, the proposed method can effectively identify pulmonary nodule.

**Table7:** Comparison of different methods for identification of pulmonary nodules.

Evaluation Criteria Method	Accuracy	Sensitivity	Specificity	F1 score
Reference [20]	0.8828	0.8382		0.8345
Reference [32]	0.85	0.85	0.85	
Reference [14]		0.8636		
This paper	0.931	0.918	0.905	0.919

## 6. Conclusion

Aiming at the problems of incomplete information expression and low detection rate of pulmonary nodules in 2D space, a new assistant diagnosis method for pulmonary nodules is proposed. After image preprocessing and segmentation, combined with the continuity information of slices, the proposed texture feature extraction method of pulmonary nodules based on volume local direction ternary pattern is used to extract features. Then the pulmonary nodules are identified by the pulmonary nodule auxiliary diagnosis model based on Stacking algorithm. This method can not only solve the loss of spatial information, but also extract texture features more comprehensively. The identification results show that this method is feasible, and can identify and classify pulmonary nodules, providing data reference for assistant diagnosis of pulmonary nodules. Nevertheless, the current method is still in semi-supervised learning state and is still limited by the accurate segmentation of pulmonary nodules. Nevertheless, the current method is still in semi-supervised learning state and is still limited by the accurate segmentation of pulmonary nodules. Therefore, future work mainly considers how to improve the diagnosis results by accurate segmentation of medical images. This paper adopts Stacking learning algorithm to identify lung nodules image. In the selection of base classifier and meta-classifier, the classifier with higher performance and recognition effect can be subsequently fused. Thus, forming a highly efficient, rapid and intelligent image-assisted diagnosis system for pulmonary nodules.

## Highlights

- A texture feature extraction algorithm for pulmonary nodules.
- Diagnosis model of pulmonary nodules based on Stacking algorithm.
- The identification of pulmonary nodules has a high accuracy rate.
